# The Effect of Night Duty of Pharmacists on Sleepiness and Concentration at Daytime

**DOI:** 10.3390/ijerph18179211

**Published:** 2021-08-31

**Authors:** Claus Werner Biechele, Martin Glos, Ingo Fietze, Jürgen Kurths, Thomas Penzel

**Affiliations:** 1 Interdisciplinary Sleep Medicine Center, Charité Universitätsmedizin Berlin, 10117 Berlin, Germany; martin.glos@charite.de (M.G.); ingo.fietze@charite.de (I.F.); kurths@pik-potsdam.de (J.K.); thomas.penzel@charite.de (T.P.); 2 Department of Medicine, The Fourth People’ Hospital of Guangyuan City, Guangyuan 628000, China; 3 Physics Department, Humboldt University, 12489 Berlin, Germany; 4 Department of Human and Animal Physiology, Saratov State University, 410012 Saratov, Russia

**Keywords:** pharmacists, night shift, concentration, actigraphy, sleepiness

## Abstract

Background: The changing responsibilities of pharmacists contribute to a lack of qualified pharmacists to fill vacant positions, particularly in rural areas. Consequently, pharmacy managers cover various duties, including an increasing number of nights being on duty that can impair daytime concentration and performance. The objective of the study was to assess the effect of night duties on daytime sleepiness, sleep quality, and concentration abilities of pharmacists. Methods: 22 pharmacists, both sexes, aged 27 to 60 years, were recruited and their sleep time, sleep efficiency, and mobility (actigraphy) were assessed during a night on duty and a control night using an actimetry. Daytime sleepiness and concentration were assessed using standardized questionnaires (ESS, KSS, d2-R). Results: Significant differences were observed between the night shift and control nights with respect to sleep time, sleep efficiency, and mobility. Daytime sleepiness was significantly increased after night shifts (ESS: 11.64 vs. 2.09; KSS: 6.77 vs. 2.41 after a night shift and control night, respectively; *p* < 0.001) and concentration diminished compared to control nights (d2-R KL: 220.95 vs. 260.36 after a night shift and control night, respectively; *p* < 0.001). Conclusions: The results provide evidence that night duties lead to high daytime sleepiness in pharmacists, which in turn may negatively affect their ability to concentrate and their error rate. Existing regulations on emergency pharmacy services should be reconsidered regarding the safety of the pharmaceutical supply.

## 1. Introduction

The shortage of pharmacies and pharmacists in Germany is reflected by a continuous decline in the number of community pharmacies during the past 20 years. Central reasons for this phenomenon are the increase in online orders of medicines and staffing difficulties due to a shift in the roles of pharmacists and unfilled positions [1]. Structurally weak rural regions are particularly affected. Likewise, the demographic change of the population with an ongoing shift of the age pyramid causes many pharmacy managers to retire, and often no replacement is found. A general trend towards part-time work and a lacking willingness to become self-employed intensify the problem. The shift of commercially relevant aspects of the medicine trade to the internet also means that pharmacists are increasingly involved in activities such as blistering, medication analysis, and laboratory work [2]. Administrative activities such as record keeping and quality management have also increased.

Pharmacy managers must therefore cover an increasing number of tasks, including more night shifts and emergency services, which means that they must work both at night and on the following day, with an overall increased number of night shifts. Usually, a night shift lasts 12 h and a day shift lasts 8–12 h. Therefore, a pharmacist on night and day duty may potentially work for 24 h in a row. Considering the responsibility of pharmacists in the preparation of prescriptions, medication analysis, verification of blistering, and care of senior citizens’ homes, this raises a relevant issue regarding the safety of medicine supply, which could be jeopardized by extended working hours of pharmacists [3]. In particular, mistakes could be made by confusing similar sounding or looking medications, miscalculating the correct dosage, or misinterpreting relevant information.

In this context, circadian periodicity is of particular importance. Physical activity and sleep behavior are linked to the synchronization of the circadian rhythm. Sleeping and waking are considered active endogenous rhythms regulated in the central nervous system. The circadian phase is of central importance for mental regeneration but also has a substantial influence on immunity, memory, and metabolism [4,5,6,7,8]. If the circadian periodicity is disturbed, for example, by night or shift work, there is a shift in sleep and wake phases and thus also a shift in physical and cognitive activity.

Significant for the evaluation of sleep deprivation in different occupational groups are the observed intra- and inter-individual differences [9]. Studies in healthcare-related occupations have demonstrated that interrupted sleep phases impact daytime sleepiness [6,10], and that concentration is impaired by lack of sleep [11,12]. James et al. noted impaired cognitive effectiveness of nurses following a 12 h night shift [11], and Thun et al. [6] detected impairments of everyday memory in nurses working night shifts. Costa et al. observed mood changes in medical residents after working all night [12], while Sanchez et al. [13] noted reduced attention and concentration ability as well as delays in the response to stimuli.

Therefore, the present study aimed to determine the impact of night work on the daytime sleepiness and concentration ability of pharmacists to identify aspects that should be considered for a change in pharmacy policy.

## 2. Materials and Methods

### 2.1. Study Design

Practicing pharmacists with night shifts were recruited into the study. As this was a pilot study, no particular inclusion or exclusion criteria were defined other than participation in night shift duties at a community pharmacy, an age lower than 60 years, and a Pittsburgh Sleep Quality Index (PSQI) value of ≤5, indicating good sleep quality for the past four weeks [14]. The study was conducted by distributing three standardized questionnaires, quantifying sleepiness and concentration ability, and an actimeter, recording rest and mobility during the night. The study procedure was the same for all participants: The control night was assessed first, followed by the night shift.

### 2.2. Actimetry

Actimetry was performed using the *MotionWatch8* (CamNtech Ltd., Cambridge, UK) worn by the pharmacists on the non-dominant wrist during one night shift and during one control night to allow for a comparison. The actimeter used was the, version 1.2.26a. Data was recorded between 10 p.m. and 8 a.m. and analyzed using MotionWatch8′s software *MotionWare* (ver. 1.2.26a, CamNtech Ltd., Cambridge, UK). The following parameters from the actimetry were evaluated: activity, mobility during the night, sleep time, sleep quality, sleep duration. Participants were asked to document the time of switching off the light and each time they got up.

### 2.3. Daytime Sleepiness and Concentration Ability

Daytime sleepiness was assessed after each night with the Karolinska Sleepiness Scale (KSS, 9-point sleepiness scale from 1 = “extremely alert” to 9 = “very sleepy”) and the Epworth Sleepiness Scale (ESS, sleepiness scale with 8 items of maximum 3 points each, 24 = maximum total score) questionnaires, while the d2-R test was employed to assess the concentration ability. KSS is a scale of situational sleepiness and measures the subjective level of sleepiness at a particular time during the day. The subject indicates which level on the scale best reflects the state during the last 10 min. The test assesses daytime sleepiness for a defined period of the previous weeks. If the test subject scores 10 or higher, a sleep medicine evaluation may be indicated. The d2-R test is used to measure concentration in tasks that require attention (focused attention). It measures the subject’s ability to concentrate and the speed and accuracy with which similar visual stimuli can be distinguished (detail discrimination).

### 2.4. Statistical Analysis

Descriptive statistics (mean, standard deviation, median, minimum, maximum) were calculated to determine sleep duration, sleep efficiency, mobility, and the questionnaire and d2R results. Means of all participants were compared with a paired t-test between the control night and the night shift. Cohen’s d was calculated to assess the practical implications of observed differences. Correlation analyses were conducted to identify factors relevant for daytime sleepiness and concentration.

## 3. Results

### 3.1. Participant Characteristics

The characteristics of the study cohort (*n* = 22) are listed in Table 1. The participants had an average age of 46.5 years (±9.62 years, range: 27–60 years) and an average PSQI of 2.18 (±0.96, range: 0–4). Seven participants (31.8%) were male. 63.6% of the participants (*n* = 14) reported no previous diseases, while one participant each (4.5%) reported previous high blood pressure, Colitis ulcerosa, mildly elevated blood pressure, neurodermitis, irritable stomach, rheumatism, dry eye, or arthritis. Eight participants did not take any medications, while one participant each (4.5%) took cortisone cream, bisoprolol, and HCT, acupuncture, or globuli. Nutritional supplements (protein shakes, glucosamine, chondroitin, magnesium, vitamin B12, salofalk, B-vitamins, zinc, selenium) were taken by one participant each (4.5%). Three of the 22 recruited participants were excluded from further assessments because Actiwatch data were not recorded for at least one night.

### 3.2. Sleep Time and Sleep Efficiency

On average, 1.82 (±1.76) prescription medicines and 5.5 (±2.89) pharmacy-only medicines were administered and 5 (±3.32) calls were answered during the night shift between 10 p.m. and 8 a.m. The sleep time and sleep efficiency were assessed within three time frames: 10 p.m. to 8 a.m., 11 p.m. to 7 a.m., and 12 a.m. to 6 a.m. The results are listed in Table 2 in comparison to the control night and the night shift. The data between the participants varied only slightly, which indicates similar sleep times and efficiencies. The difference between the night shift and the control night decreased more the smaller the time window was. For example, between 10 p.m. and 8 a.m., the participants slept on average about 72 min less on night duty than in the control night. In the 24 to 6 a.m. period, this difference decreased to an average of about 20 min. The same pattern was observed with sleep efficiency, where the difference between night duty and control night also decreased as the time window was decreased. Interestingly, the sleep efficiency increased overall as the time window decreased, both during the night shift and in the control night. Table 3 lists the statistical evaluation of differences in the sleep quality and efficiency between the control night and the night shift. The mean sleep time during the night shift was significantly less than the sleep time during the control night for the time windows of 10 p.m. to 8 a.m. (*p* < 0.001) and 11 p.m. and 7 a.m. (*p* < 0.01). The differences between the shortest time window of 12 a.m. to 6 a.m. did not significantly differ (*p* = 0.071). Sleep efficiency significantly differed between the night shift and the control night for all time windows (Table 3). Cohen’s d was calculated to determine the practical significance of these statistically significant differences calculated with the t-test. For the time window of 10 p.m. to 8 a.m., Cohen’s d for the sleep time was −1.30, for the time window 11 p.m. to 7 a.m. −1.09, and for the smallest time window of 12 a.m. to 6 a.m. it was −0.61. Therefore, Cohen’s d surpassed the critical value of 0.57 for all three time periods assessed [15] (Table 3). The same was found for differences in sleep efficiency.

### 3.3. Mobility during the Night

Mobile and immobile minutes for the control night and the night shift were compared based on the data of the Actiwatch (Table 2). For the time window of 10 p.m. to 8 a.m., the number of immobile minutes amounted to 322.6 (±50.1 min) during the night shift and to 391.7 (±53.4 min) during the control night. The number of mobile minutes during the night shift exceeds those during the control night, respectively (night shift: 84 ± 36.3 min; control night: 63.8 ± 38.9 min). The same was true for both shorter time windows, with less immobile minutes and more mobile minutes counted during the night shift compared to the control night. The observed differences in the immobile minutes were significant for all three time windows (10 p.m. to 8 a.m.: *p* < 0.001; 11 p.m. to 7 a.m.; *p* < 0.001; 12 a.m. to 6 a.m.; *p* < 0.05), while no statistically significant differences were observed for the mobile minutes (10 p.m. to 8 a.m.: *p* = 0.132; 11 p.m. to 7 a.m.; *p* = 0.146; 12 a.m. to 6 a.m.; *p* = 0.051; Table 3). Cohen’s d for the differences in the immobile minutes exceeded the critical value of 0.57 for all three time windows.

### 3.4. Daytime Sleepiness

Both the ESS and the KSS revealed significantly higher scores after a night shift compared to a control night (Table 4 and Table 5). Average ESS scores amounted to 11.64 ± 3.05 after a night on duty and to 2.09 ± 1.87 after a control night (*p* < 0.001). Similarly, an average KSS score of 6.77 ± 0.81 was determined after a night shift, while the average score after a control night was 2.41 ± 0.59 (*p* < 0.001). Cohen’s d exceeded the critical value for both tests (ESS: 3.81; KSS: 6.19).

### 3.5. Concentration

Five parameters related to concentration were analyzed with the d2-R tests, the mistake rate (F%), the concentration ability (KL), the mistake of mixing up objects within the test (VF), the mistake of leaving out correct objects (AF), and the number of marked target objects within the allowed test time (BZO) (Table 4). The three parameters identifying mistakes (F%, AF, and VF) were significantly higher after a night shift compared to a control night, while significantly fewer objects were marked within the test time (BZO) and the concentration ability was significantly lower after a night shift (KL; *p* < 0.001 for all d2-R parameters, Table 5). Cohen’s d indicated a strong practical implication of all five d2-R parameters.

### 3.6. Correlations

A high correlation between sleep time, sleep efficiency and the number of immobile minutes was detected in the correlation analysis in both the control night and the night shift and in all three time periods assessed (Table 6). If sleep time was used to represent also sleep efficiency and immobile minutes, the correlation with daytime sleepiness and concentration was low in the control night. In contrast, higher correlations pertaining to daytime sleepiness and concentration were observed. The longer the sleep time during the night shift, the lower the VF on the concentration scale was the next day, indicating that less objectives were falsely mixed up in the d2-R-test. In turn, the shorter a participant slept, the worse his or her concentration ability was on the next day.

## 4. Discussion

For the first time, we identified an impact of night shift work of pharmacists on their sleep duration and sleep efficiency. Compared to a control night, pharmacists were mobile for longer periods and demonstrated an increased daytime sleepiness on the following day. Furthermore, pharmacists showed a diminished concentration ability after a night shift that correlated with daytime sleepiness.

The continuous closure of pharmacies renders pharmacy managers in Germany responsible for numerous tasks and increases the number of nights they are on duty. At the same time, they frequently continue work after a night shift, which may impair their concentration and hence the safe distribution of medicines. In the present study, the impact of night duties on daytime sleepiness and concentration ability was assessed.

The results show that pharmacists on night duty slept considerably less and with a reduced efficiency during nights on duty compared to control nights. This significantly impaired their concentration ability during the day and increased their daytime sleepiness. Significant differences between the control night and the night on duty were observed for all three time frames and for three of the four parameters evaluated (sleep time, sleep efficiency, immobile minutes), while the number of mobile minutes did not differ between both nights. Particularly, the fact that the concentration was significantly impaired after a night shift is of concern, considering the responsibility the pharmacists have regarding blistering, fulfillment of prescriptions, and laboratory analyses. The impairment of concentration after a night shift has been documented for several other health care professions and concerns for the wellbeing of these professional groups and the safety of the patients have been raised [11,12,16,17,18].

German legislation requires pharmacies to be on call at all times to ensure the constant supply of medicines. This applies on working days, weekends and public holidays and is intended to secure the supply, especially when other sectors of the economy are at rest. The pharmacy on-call service is not regarded as a way for pharmacies to operate economically outside of store opening hours, but rather as an emergency service that guarantees the population rapid access to medicines. This on-call service is normally permanent, although it is up to the competent authority to exempt a certain proportion of pharmacies from this on-call duty during certain periods. If there is no exemption, the pharmacy manager or a person authorized to represent him must be always available during the on-call hours. The pharmacy manager can be represented by a pharmacist, but this is only possible if this person is a permanent substitute and a substitution is, therefore, the exception.

Based on the results of the present study, the need to address this issue in pharmacy policy and legislation is obviated. The goal of the health authority is to ensure a uniform exemption for pharmacies. However, this does not always prove possible in practice. Strategies to determine and alleviate the on-duty hours of pharmacy managers appear essential in improving their wellbeing while ensuring the safety of the customers. Importantly, the suitability of an actimeter to assess the sleep duration, efficiency, and mobility during a night on duty was demonstrated herein and hence it could offer a valuable tool in analyzing these factors for healthcare workers on night shifts. Moreover, policies to attract young pharmacists to rural areas and support their self-employment as community pharmacists are indicated.

The present study has certain limitations that should be addressed. First, it must be considered a pilot study due to the low number of participants. Therefore, no inclusion and exclusion criteria were defined but should define the cohort of future studies. Second, only one region of Germany was assessed, which should be extended to a more representative sample with pharmacists from several geographical regions. Third, data was collected for each participant during and after a control night and a night shift. Last, the ESS and KSS questionnaires are subjective measures of daytime sleepiness and hence the results obtained using these questionnaires may not be generally extrapolated. Nonetheless, the d2R questionnaire offers an objective measure, and hence the results are considered less subjective and more reliably transposable. Although the d2-R has not been specifically validated for pharmacists, it is a standard tool to assess concentration ability. The items assessed, particularly counting and distinguishing objects under time pressure, are relevant for the pharmacists, i.e., in counting pills and distinguishing pills with a similar appearance. In addition, the ESS scores of >10 are comparable with previous studies on sleepiness after a night shift in nurses [19], medical residents [20,21], and emergency physicians [22]. Last, the circadian tendency of participants was not taken into account due to the small sample size. It is feasible to assume that participants with eveningness tendencies may perform better on night shifts compared to those with a morning tendency. The results of the present pilot study indicate that future studies on larger cohorts are worth assessing the impact of night duty on daytime sleepiness and identify policies to improve workplace safety of pharmacists and other healthcare workers.

## 5. Conclusions

The present pilot study gives an indication of the negative impact of extensive night shift work on daytime sleepiness and performance and as a consequence safety of medication distribution. Due to the limitations of the study, future studies should investigate the impact of night shifts in a larger cohort of pharmacists and ideally during several consecutive night shifts to exclude any confounders such as incidental events during one night shift and personal situations of the few pharmacists investigated herein.

## Figures and Tables

**Table 1:** Participant characteristics (*n* = 22). PSQI, Pittsburgh Sleep Quality Index; SD, standard deviation; BMI, Body Mass Index. PSQI, body weight, height, and BMI were measured before the control night, the age was determined before the night shift.

Variable	Mean	SD	Median	Minimum	Maximum
PSQI	2.18	0.96	2	0	4
Age	46.45	9.62	45.50	27	60
Body weight (kg)	72	16.05	68.50	52	102
Height (m)	1.72	0.09	1.70	1.59	1.91
BMI (kg/m²)	24.24	4.29	23.47	18.91	37.18

**Table 2:** Sleep time, sleep efficiency, mobile/immobile minutes, during the control night and the night shift (n total = 38, n night shift = 19, n control night = 19). SD, standard deviation.

Variable	Time	Type of Night	Mean	SD	Median	Minimum	Maximum
Sleep time	10 p.m. to 8 a.m.	Total	323.53	65.70	316.50	168	468
		Night shift	287.32	48.55	287	168	361
		Control night	359.74	61.21	361	257	468
	11 p.m. to 7 a.m.	Total	270.37	50.44	265	146	375
		Night shift	246	43.07	253	146	301
		Control night	294.74	46.06	284	217	375
	12 a.m. to 6 a.m.	Total	211.50	35.01	207.50	120	282
		Night shift	201.16	32.41	203	120	252
		Control night	221.84	35.27	215	173	282
Sleep efficiency	10 p.m. to 8 a.m.	Total	67.41	13.68	65.95	35	97.50
		Night shift	59.86	10.11	59.80	35	75.20
		Control night	74.95	12.74	75.20	53.50	97.50
	11 p.m. to 7 a.m.	Total	70.40	13.14	69.10	38	97.70
		Night shift	64.05	11.21	65.90	38	78.40
		Control night	76.76	12	74	56.50	97.70
	12 a.m. to 6 a.m.	Total	73.78	11.96	72.70	41.80	97.90
		Night shift	69.88	11.28	70.50	41.80	87.50
		Control night	77.68	11.62	74.70	62.50	97.90
Immobile minutes	10 p.m. to 8 a.m.	Total	357.16	61.92	353	201	476
		Night shift	322.58	50.05	328	201	405
		Control night	391.74	53.39	395	291	476
	11 p.m. to 7 a.m.	Total	297.82	47.33	306	173	381
		Night shift	273.63	45.14	285	173	338
		Control night	322	36.51	322	247	381
	12 a.m. to 6 a.m.	Total	232.50	31.30	235	143	286
		Night shift	223.05	32.66	231	143	267
		Control night	241.95	27.55	240	193	286
Mobile minutes	10 p.m. to 8 a.m.	Total	73.92	38.51	73	4	176
		Night shift	84	36.28	73	43	176
		Control night	63.84	38.95	71	4	155
	11 p.m. to 7 a.m.	Total	55.97	25.81	58	3	107
		Night shift	61.63	22.92	57	23	107
		Control night	50.32	27.87	59	3	102
	12 a.m. to 6 a.m.	Total	42.55	20.45	43	2	95
		Night shift	48.11	16.17	52	19	81
		Control night	37	23.08	36	2	95

**Table 3:** Comparison of sleep time, sleep efficiency, and mobile/immobile minutes between the control night and the night shift. CI, confidence interval; SD, standard deviation.

Variable	Time	Mean (SD)			t	*p*	95% CI	Cohen’s d
		Night Shift	Control Night	Difference				
Sleep time	10 p.m. to 8 a.m.	287.32 (48.55)	359.74 (61.22)	−72.42 (61.86)	−5.10	<0.001	[−102.24; −42.61]	−1.30
	11 p.m. to 7 a.m.	246 (43.07)	294.74 (46.06)	−48.74 (58.68)	−3.62	0.002	[−77.01; −20.46]	−1.09
	12 a.m. to 6 a.m.	201.16 (32.41)	221.84 (35.27)	−20.68 (46.97)	−1.92	0.071	[−43.32; 1.95]	−0.61
Sleep efficiency	10 p.m. to 8 a.m.	59.86 (10.11)	74.95 (12.74)	−15.08 (12.88)	−5.10	<0.001	[−21.29; −8.87]	−1.30
	11 p.m. to 7 a.m.	64.05 (11.21)	76.76 (12)	−12.71 (15.26)	−3.63	0.002	[−20.06; −5.36]	−1.09
	12 a.m. to 6 a.m.	69.88 (11.28)	77.68 (11.62)	−7.8 (16.04)	−2.12	0.048	[−15.53; −0.07]	−0.68
Immobile minutes	10 p.m. to 8 a.m.	322.58 (50.05)	391.74 (53.39)	−69.16 (47.30)	−6.37	< 0.001	[−91.95; −46.36]	−1.33
	11 p.m. to 7 a.m.	273.63 (45.14)	322 (36.51)	−48.37 (48.23)	−4.37	< 0.001	[−71.62; −25.12]	−1.17
	12 a.m. to 6 a.m.	223.05 (32.66)	241.95 (27.55)	−18.89 (38.30)	−2.15	0.045	[−37.36; −0.43]	−0.62
Mobile minutes	10 p.m. to 8 a.m.	84 (36.28)	63.84 (38.95)	20.16 (55.69)	1.58	0.132	[−6.68; 47]	0.54
	11 p.m. to 7 a.m.	61.63 (22.92)	50.32 (27.87)	11.32 (32.45)	1.52	0.146	[−4.32; 26.96]	0.44
	12 a.m. to 6 a.m.	48.11 (16.17)	37 (23.08)	11.11 (23.19)	2.09	0.051	[−0.07; 22.28]	0.55

**Table 4:** Daytime sleepiness and concentration ability after the control night, the night shift, and in total. AF, “Auslassungsfehler“/omissions, BZO, “bearbeitete Zielobjekte”/number of completed objects; CI, confidence interval; ESS, Epworth Sleep Scale; F%, „Fehlerquote“/mistake rate; KL, “Konzentrationsleistung”/concentration, SD, standard deviation; VF, “Verwechslungsfehler”/mix-up mistakes.

Variable		Type of Night	Mean	SD	Median	Minimum	Maximum
ESS		Total	6.86	5.44	6.50	0	17
		Night shift	11.64	3.05	12	4	17
		Control night	2.09	1.87	2	0	7
Karolinska		Total	4.59	2.32	5	2	9
		Night shift	6.77	0.81	7	6	9
		Control night	2.41	0.59	2	2	4
d2-R	BZO	Total	259.02	24.52	259.50	204	301
		Night shift	245.68	22.37	251.50	204	301
		Control night	272.36	18.93	276.50	208	299
	AF	Total	12.55	9.33	10	1	46
		Night shift	15.95	10.41	14	2	46
		Control night	9.14	6.76	6	1	28
	VF	Total	5.82	4.47	5	0	17
		Night shift	8.77	4.19	9	0	17
		Control night	2.86	2.27	2.50	0	7
	KL	Total	240.66	28.11	244	180	282
		Night shift	220.95	21.41	228	180	248
		Control night	260.36	18.59	266	207	282
	F%	Total	7.15	4.73	6.70	0.10	21
		Night shift	9.95	4.79	8.75	2.40	21
		Control night	4.35	2.54	4.05	0.10	11

**Table 5:** Comparison of daytime sleepiness and concentration ability between the control night and the night shift. AF, “Auslassungsfehler“/omissions, BZO, “bearbeitete Zielobjekte”/number of completed objects; CI, confidence interval; ESS, Epworth Sleep Scale; F%, “Fehlerquote”/mistake rate; KL, “Konzentrationsleistung”/concentration, SD, standard deviation; VF, “Verwechslungsfehler”/mix-up mistakes.

Variable		Mean (SD)			t	*p*	95% CI	Cohen’s d
		Night Shift	Control Night	Difference				
ESS		11.64 (3.05)	2.09 (1.87)	9.55 (3.94)	11.37	<0.001	[7.80; 11.29]	3.81
Karolinska		6.77 (0.81)	2.41 (0.59)	4.36 (1.18)	17.39	<0.001	[3.84; 4.89]	6.19
d2-R	BZO	245.68 (22.37)	272.36 (18.93)	−26.68 (15.64)	−8.00	<0.001	[−33.62; −19.75]	−1.27
	AF	15.95 (10.41)	9.14 (6.76)	6.82 (6.40)	4.99	<0.001	[3.98; 9.66]	0.67
	VF	8.77 (4.19)	2.86 (2.27)	5.91 (3.78)	7.34	<0.001	[4.23; 7.58]	1.65
	KL	220.95 (21.41)	260.36 (18.59)	−39.41 (15.71)	−11.77	<0.001	[−46.37; −32.44]	−1.94
	F%	9.95 (4.79)	4.35 (2.54)	5.60 (3.57)	7.37	<0.001	[4.02; 7.19]	1.25

**Table 6:** Pearson correlation matrix of sleep, daytime sleepiness, and concentration during the night shift. AF, “Auslassungsfehler“/omissions, BZO, “bearbeitete Zielobjekte”/number of completed objects; CI, confidence interval; ESS, Epworth Sleep Scale; F%, “Fehlerquote”/mistake rate; KL, “Konzentrationsleistung”/concentration, SD, standard deviation; VF, “Verwechslungsfehler”/mix-up mistakes. ** *p* < 0.01.

Variable		Sleep Time			Sleep Efficiency			d2-R					ESS
		10 p.m. to 8 a.m.	11 p.m. to 7 a.m.	12 a.m. to 6 a.m.	10 p.m. to 8 a.m.	11 p.m. to 7 a.m.	12 a.m. to 6 a.m.	BZO	AF	VF	KL	F%	
Sleep time	11 p.m. to 7 a.m.	0.95 **											
		[0.87. 0.98]											
	0 a.m. to 6 a.m.	0.86 **	0.93 **										
		[0.67. 0.95]	[0.82. 0.97]										
Sleep efficiency	10 p.m. to 8 a.m.	1.00 **	0.95 **	0.86 **									
		[1.00. 1.00]	[0.87. 0.98]	[0.67. 0.95]									
	11 p.m. to 7 a.m.	0.95 **	1.00 **	0.93 **	0.95 **								
		[0.88. 0.98]	[1.00. 1.00]	[0.82. 0.97]	[0.88. 0.98]								
	0 a.m. to 6 a.m.	0.86 **	0.93 **	1.00 **	0.86 **	0.93 **							
		[0.67. 0.95]	[0.82. 0.97]	[1.00. 1.00]	[0.67. 0.95]	[0.83. 0.97]							
d2-R	BZO	−0.34	−0.24	−0.12	−0.33	−0.24	−0.12						
		[−0.69. 0.14]	[−0.63. 0.24]	[−0.55. 0.35]	[−0.68. 0.14]	[−0.63. 0.24]	[−0.55. 0.35]						
	AF	0.13	0.20	0.17	0.13	0.20	0.17	0.20					
		[−0.34. 0.55]	[−0.28. 0.60]	[−0.31. 0.58]	[−0.34. 0.55]	[−0.28. 0.60]	[−0.31. 0.58]	[−0.28. 0.60]					
	VF	−0.59 **	−0.40	−0.32	−0.59 **	−0.41	−0.32	0.36	0.17				
		[−0.82. −0.18]	[−0.73. 0.06]	[−0.67. 0.16]	[−0.82. −0.18]	[−0.73. 0.06]	[−0.68. 0.16]	[−0.11. 0.70]	[−0.31. 0.58]				
	KL	−0.30	−0.27	−0.15	−0.30	−0.27	−0.15	0.86 **	−0.31	0.11			
		[−0.66. 0.18]	[−0.64. 0.21]	[−0.57. 0.33]	[−0.66. 0.18]	[−0.64. 0.21]	[−0.57. 0.32]	[0.66. 0.94]	[−0.67. 0.17]	[−0.36. 0.54]			
	F%	−0.01	0.10	0.08	−0.01	0.10	0.08	0.05	0.92 **	0.44	−0.47 *		
		[−0.46. 0.45]	[−0.37. 0.53]	[−0.38. 0.52]	[−0.46. 0.45]	[−0.38. 0.53]	[−0.38. 0.52]	[−0.42. 0.49]	[0.80. 0.97]	[−0.02. 0.75]	[−0.76. −0.02]		
ESS		−0.22	−0.15	−0.06	−0.22	−0.15	−0.05	−0.05	0.41	0.44	−0.33	0.59 **	
		[−0.61. 0.26]	[−0.57. 0.33]	[−0.50. 0.41]	[−0.61. 0.26]	[−0.57. 0.33]	[−0.49. 0.41]	[−0.50. 0.41]	[−0.05. 0.73]	[−0.02. 0.74]	[−0.68. 0.15]	[0.18. 0.82]	
Karolinska		−0.40	−0.25	−0.26	−0.40	−0.25	−0.26	0.15	0.45	0.61 **	−0.17	0.58 **	0.58 **
		[−0.72. 0.06]	[−0.63. 0.23]	[−0.64. 0.22]	[−0.72. 0.06]	[−0.63. 0.23]	[−0.64. 0.22]	[−0.32. 0.57]	[−0.00. 0.75]	[0.21. 0.83]	[−0.58. 0.31]	[0.17. 0.82]	[0.16. 0.82]

## Data Availability

The data presented in this study are available on request from the corresponding author.
